# Environmental physiotherapy: knowledge, attitudes, and practices among physiotherapists in Iceland

**DOI:** 10.3389/fpubh.2025.1528217

**Published:** 2025-02-13

**Authors:** Steinunn A. Olafsdottir, Lilja Salome H. Petursdottir

**Affiliations:** ^1^Department of Physiotherapy, Faculty of Medicine, University of Iceland, Reykjavik, Iceland; ^2^Landspitali, National University Hospital in Reykjavik, Reykjavik, Iceland

**Keywords:** climate change, physiotherapy, health education and awareness, environmental impact, health promotion

## Abstract

**Introduction:**

With growing concerns about climate change and the healthcare sector's carbon footprint, integrating sustainable practices into physiotherapy could not only reduce this impact but also enhance patient outcomes. This study explores the knowledge, attitudes, and practices of physiotherapists in Iceland regarding climate change and their role in promoting sustainability.

**Methods:**

A cross-sectional survey was distributed to active members of the Icelandic Physiotherapy Association. A total of 114 physiotherapists participated (17.1% response rate). The survey, consisting of 21 questions across four themes—knowledge, attitudes, behavior, and obstacles—captured insights on respondents' understanding of climate change, their environmental practices, and the challenges they encounter.

**Results:**

The results revealed a strong awareness of the relevance of climate change to health, though many respondents acknowledged limited knowledge on the topic. A majority expressed a sense of responsibility to mitigate climate change and reported taking actions such as reducing waste and promoting energy conservation. However, many identified a need for more guidance and training to integrate sustainable practices effectively. The primary obstacle was insufficient knowledge on implementing environmental strategies, with respondents indicating that education and resources would help overcome this barrier.

**Discussion:**

This study highlights the potential for physiotherapists to contribute significantly to the healthcare sector's sustainability goals. The respondents' desire to enhance their environmental practices suggests an opportunity for professional associations and educational institutions to provide targeted training and support. By fostering greater environmental literacy, physiotherapists could not only contribute to reducing the healthcare sector's carbon footprint but also promote sustainable health behaviors in patients. Enhanced knowledge and support could help physiotherapists become key contributors to sustainability in healthcare.

## 1 Introduction

The World Health Organization has identified climate change as the greatest health threat facing humanity (1). The connection between climate change and human health has been well-studied, revealing a dual challenge for healthcare systems. Firstly, rising temperatures and extreme weather events have been linked to various health issues, including respiratory and cardiovascular diseases, mental health disorders, and malnutrition with increased burden on healthcare systems (2). Secondly, the healthcare sector itself contributes significantly to environmental problems, accounting for up to 5% of global negative environmental impacts (3). Globally, it accounts for substantial greenhouse gas emissions, comparable to the emissions of 514 coal-fired power plants (4). Most of these emissions come from the supply chain, including the production, transportation, and disposal of medical goods and services.

A coordinated effort from all healthcare professions is essential to reduce the carbon footprint of the healthcare system (5). Physiotherapists can play a significant role, provided they have the necessary knowledge and understanding of how climate issues relate to their work (6). Physiotherapy inherently supports environmental sustainability through non-invasive treatments that can reduce the need for surgeries and medication (7, 8). Studies also suggest that encouraging active transportation among patients can simultaneously benefit their health and the environment (9).

International research indicates that healthcare professionals are generally aware of climate issues and their impact on health but often lack the detailed specific knowledge and practical tools needed to address these issues effectively (10–14). However, very few research have had specific focus on physiotherapists. A study from Spain (15) on physiotherapy students, indicated that the students were generally aware of climate issues and acknowledged their potential relevance to their future profession, but their knowledge was still somewhat lacking, and they did not perceive their studies to have adequately addressed the issue.

This study is the first to examine the knowledge, attitudes, and practices of physiotherapists in Iceland regarding climate issues, a topic largely unexplored in the field of healthcare. While previous research has highlighted the broader environmental impacts of healthcare systems, little attention has been given to the specific contributions and challenges within physiotherapy. This study is not only a contribution to fill this critical gap but also provides a unique perspective by focusing on Iceland—a country with a distinctive healthcare system and a strong commitment to sustainability. Furthermore, it explores how climate-conscious physiotherapy practices can positively impact public health by promoting environmentally sustainable healthcare delivery. By addressing these under-researched intersections, the study provides novel insights that not only advance physiotherapy practice but also align with global efforts to mitigate climate change and enhance population health outcomes.

Our aim is to answer the following research questions:

- What is the level of awareness among physiotherapists in Iceland regarding the health impacts of climate change?
- How do physiotherapists in Iceland perceive the relevance of climate change to their professional roles and responsibilities?
- What eco-friendly practices have physiotherapists in Iceland adopted to address climate issues in their work?
- What barriers and facilitators influence the integration of climate issues into physiotherapy practice in Iceland?

## 2 Methods

### 2.1 Study design and survey development

A cross-sectional survey was designed to gather data from physiotherapists in Iceland. The survey was developed and adapted from existing literature (10, 11, 13, 14) consisted of 17 questions divided into four main themes: knowledge, attitudes, behavior/practice, and potential obstacles and facilitators for eco-friendly practice.

1. Knowledge: Four questions aimed at assessing the respondents' understanding of climate change and its relevance to physiotherapy and health.
2. Attitudes: Seven statements focused on the respondents' beliefs about the role of physiotherapists in addressing climate change and their perceived importance of this issue. Participants were asked to express their attitudes toward these statements on a 5-point Likert scale.
3. Behavior/practice: Four questions on specific actions respondents were already taking to increase sustainability and reduce their carbon footprint in their professional practice as well as personal choices.
4. Obstacles and facilitators: Two questions on perceived obstacles respondents were facing in adopting eco-friendly practices and the types of support or incentives that would be most helpful in overcoming these barriers. Participants marked potential obstacles and facilitators from a list.

### 2.2 Participants

The survey was sent via email to all active members of The Icelandic Physiotherapy Association and was also promoted in a Facebook group for physiotherapists in Iceland. The background questions included questions on gender, age-group (10 years interval from 25 to 75 years old) and workplace setting, participants were informed about the study's purpose, their right to withdraw, and the confidentiality of their responses. Data was anonymized to ensure privacy.

### 2.3 Statistical analysis

Descriptive statistics summarized demographics, knowledge, attitudes, and practices. Chi-square tests and *t*-tests were used to examine associations between demographic variables and respondents' knowledge, attitudes, and practices. Thematic analysis (16) was used for qualitative data from open-ended responses.

## 3 Results

A total of 114 participants responded to the questionnaire. Most of participants were women, and the number was equal across all age groups from 25 to 65 years (Table 1). Approximately half of the participants worked in private clinics, little < 40% in institutions (hospitals and rehabilitation centers), and around 12% work in teaching, research, or other fields.

**Table 1:** Demography of participants.

		**Number (%)**
Sex	Female	93 (82.3%)
	Male	19 (16.8%)
	Other/prefer not to answer	1 (0.9%)
Age	25–35 years	31 (27.2%)
	36–45 years	26 (22.8%)
	46–55 years	24 (21.1%)
	56–65 years	28 (24.6%)
	66–75 years	5 (4.4%)
Workplace setting	Private clinics	57 (50%)
	Institutions	43 (37.7%)
	Teaching/research	2 (1.8%)
	Other, unspecified	12 (10.5%)

### 3.1 Knowledge of climate change and relation to health

Most participants (56.6%, *n* = 64) assessed their own knowledge of climate issues as intermediate. However, a total of 38.1% rated their knowledge as advanced (*n* = 3) or rather advanced (*n* = 40) and 5.3% (*n* = 6) rated their knowledge as rather little while no participant reported having very little knowledge.

**Figure 1:**
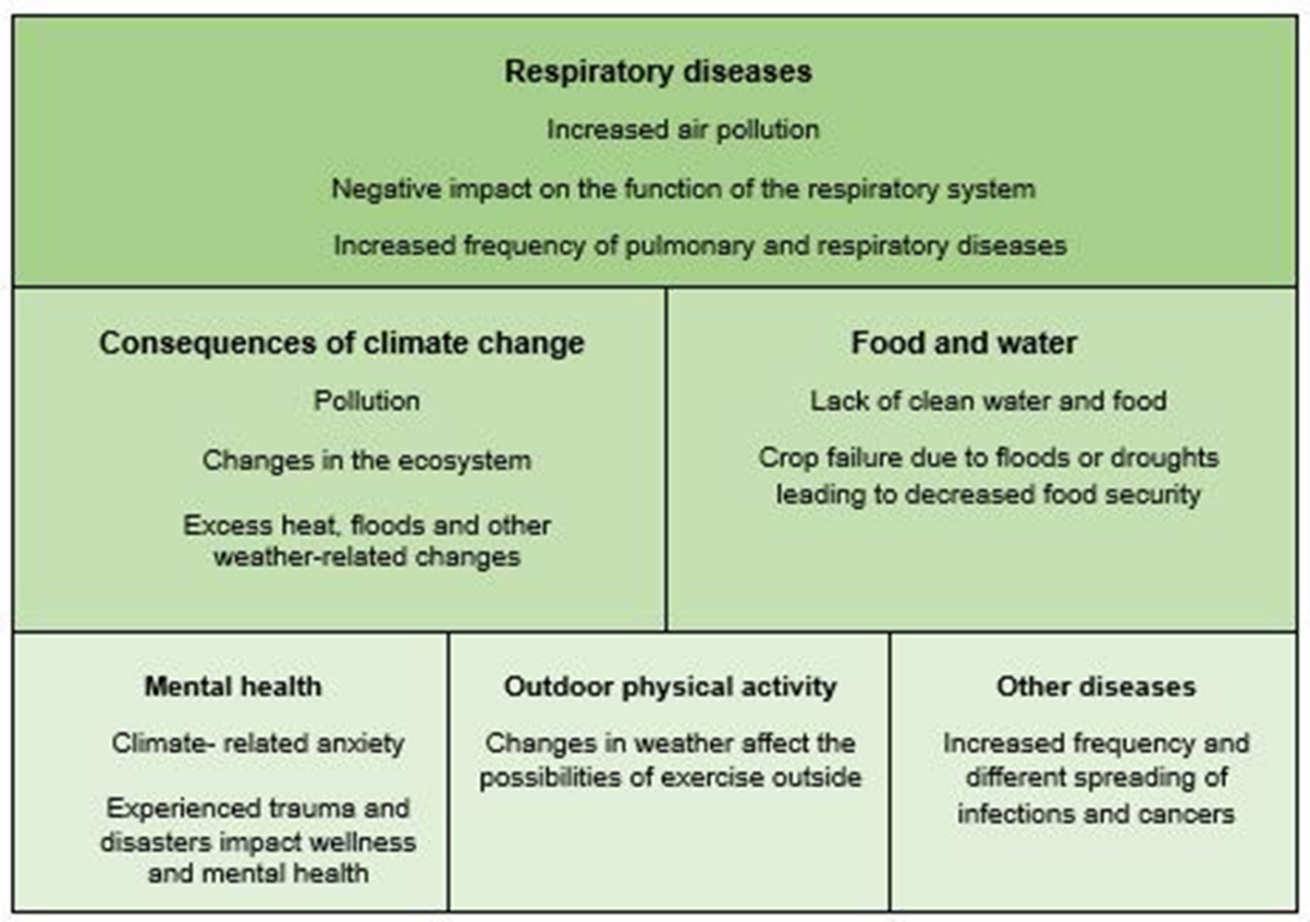
Themes on how climate change affects health.

There was a significant difference (*p* < 0.001) in how many considered climate change to affect people's health in Iceland compared to other countries. In total, 84.1% (*n* = 95) of participants believed that climate change and its consequences affect people's health in Iceland, either directly or indirectly while 97.3% (*n* = 108) believed the same applies outside Iceland. When asked, in an open question, how they believed climate change or its consequences affect people's health, 84 participants wrote responses, from which several main themes were derived (Figure 1).

Air pollution and respiratory diseases were prominent in the responses, with many participants linking the two together and indicating that increasing air pollution negatively affects lung and respiratory function, potentially leading to an increased frequency of pulmonary and respiratory diseases. Consequences of climate change were also often mentioned, although not everyone connected them to health effects. References were made to pollution, changes in the Earth's biodiversity and oceans, heat, floods, and other weather-related changes, although it was not always clear how these changes would affect people's health. For example, one participant simply wrote: “air pollution, weather changes, ocean pollution” while another one gave a more detailed response:

“For example, water shortage, leading to crop failures, hunger, malnutrition, and death. More extreme weather resulting in damage to housing, increased risk of epidemics, disrupted transportation, and many people get displaced and suffer. Increased risk of floods and thereby destruction of valuables and crop failures. This, and much more, obviously has very negative effects on the health of those affected. Global warming and rising sea levels affect both marine and terrestrial ecosystems, which affects residents.”

In some cases, accidents due to extremes in weather were also mentioned.

Many participants reported the effects on water and food supply, addressing both the lack of access to clean and unpolluted water and food as well as crop failures due to floods and droughts, which could affect food security. One participant wrote:

“Significant changes in the weather can cause crop failures and affect food security, which in turn can cause illness or even death. Water shortage or the opposite, floods, have very serious consequences, as many examples from the world in recent years show.”

Mental health was also often addressed. This included discussion of so-called climate anxiety and concerns about the future. “Will it be habitable in the future, will my children or grandchildren be okay?” is an example of one participant's reflection. Additionally, the impact of experiencing natural disasters and traumas related to changing living conditions on mental wellbeing was also reflected. One participant mentioned “effects on the nervous system (e.g., stress, pressure from losing housing or family members),” while another noted that the effects of climate change can “cause anxiety and insecurity, which leads to reduced quality of life regarding both mental and physical health.”

Several participants mentioned the impact of weather changes on the ability to exercise outdoors. “Worse weather = less outdoor activity = less exercise,” one participant simply stated. Another participant reported: “Extreme weather conditions, in either direction, make people potentially exercise less outdoors, with overall negative effects on their quality of life”. Yet another pointed out the effect of limited outdoor activity on mental and physical health:

“People go outside less often if there is more extreme weather or precipitation due to climate change, which negatively impacts mental and physical health.”

Finally, a few participants mentioned other diseases, such as the increased frequency and changing distribution of infectious diseases, cancer, and the impact of weather on the symptoms of individuals with arthritis, although it was not always clear in what way they believed climate change affected these factors.

### 3.2 Attitudes toward climate change and role of physiotherapists

Majority of participants (83%, *n* = 88) strongly agreed or agreed that the negative environmental impact of healthcare services needs to be limited, and similar number (80.5%, *n* = 87) agreed or strongly agreed that the negative environmental impact of physiotherapy services should be minimized (Table 2). Additionally, 78.7% of participants (*n* = 85) were very or somewhat concerned about climate change, while 8.3% (*n* = 9) were somewhat or strongly opposed to this statement. Most participants (87%, *n* = 84) were interested in learning more about how they could reduce environmental impacts in their work. In total, 66.7% of participants (*n* = 72) strongly agreed or agreed that physiotherapists should raise public awareness of the health impacts of climate change. As many (*n* = 72) felt that physiotherapists should make their clients aware of the health effects of climate change, although a little more strongly agreed on that statement. Finally, a total of 68.2% (*n* = 73) strongly agreed or agreed that physiotherapy is inherently eco-friendly while 27.1% (*n* = 29) were neutral on the statement.

**Table 2:** Attitudes toward climate change and role of physiotherapists.

	**Number (%)**				
**Statement**	**Strongly agree**	**Agree**	**Neither agree nor disagree**	**Disagree**	**Strongly disagree**
It is necessary to limit the negative impact of healthcare services on the environment	47 (44.3%)	41 (38.7%)	13 (12.3%)	2 (1.9%)	3 (2.8%)
The negative impact of physiotherapy services on the environment must be limited	46 (42.6%)	41 (38%)	14 (13%)	2 (1.9%)	5 (4.6%)
I am concerned about climate change	31 (28.7%)	54 (50%)	14 (13%)	4 (3.7%)	5 (4.6%)
I am interested in learning more how I can reduce environmental impact in my work	53 (49.1%)	41 (38%)	10 (9.3%)	1 (0.9%)	3 (2.8%)
Physiotherapists should raise public awareness of the health impacts of climate change	21 (19.4%)	51 (47.2%)	21 (19.4%)	7 (6.5%)	8 (7.4%)
Physiotherapists should raise their clients' awareness of the health impacts of climate change	27 (25%)	45 (41.7%)	23 (21.3%)	8 (7.4%)	5 (4.6%)
Physiotherapy is inherently eco-friendly	29 (27.1%)	44 (14.1%)	29 (27.1%)	3 (2.8%)	2 (1.9%)

**Figure 2:**
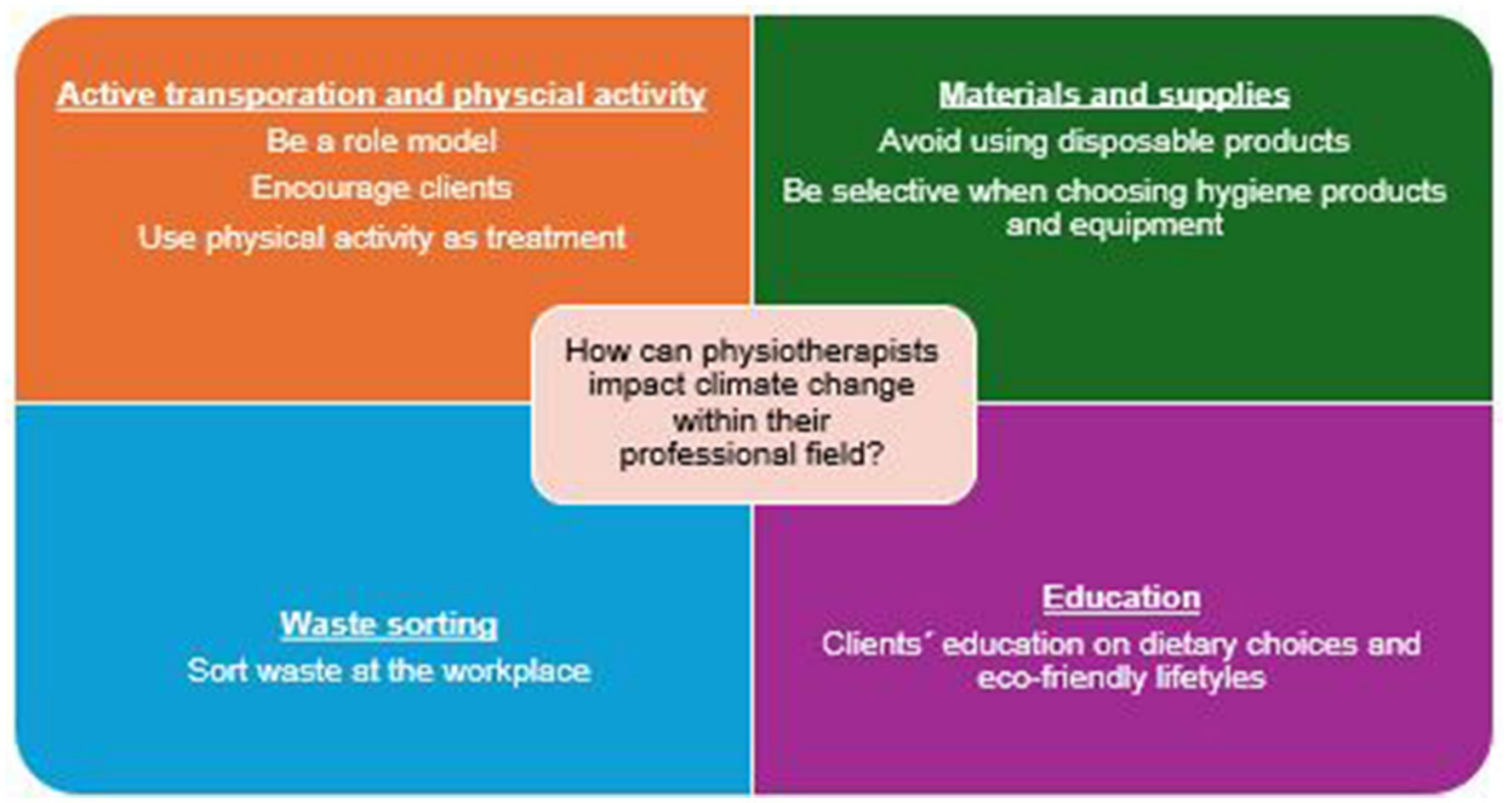
Examples on how physiotherapists can impact climate change.

When asked whether participants believed that physiotherapists could have an impact (either negative or positive) on climate change within their professional field, most participants (79.6%, *n* = 82) agreed. A total of 60 participants responded to an open-ended question, providing examples on how they thought physiotherapists could reduce the impact on climate health within their workplace setting. Four themes were identified from their answers (Figure 2).

The first theme, and the most frequently mentioned, was active transportation and exercise. Participants primarily suggested that physiotherapists could encourage their clients to use active transportation instead of private cars to improve both their quality of life and health while reducing environmental impact. Additionally, participants highlighted the importance of being role models in this regard. One participant stated that physiotherapists could “encourage and set an example by using self-propelled transportation, such as choosing stairs over elevators, walking instead of driving, etc.” Exercise was also cited as an eco-friendly form of therapy.

The second theme focused on materials and supplies, with participants mentioning reducing the use of disposable products and paper and being selective with hygiene products and other equipment. One participant recommended “conscious use of equipment and supplies, such as choosing reusable over disposable items.” The third theme concerned waste sorting, specifically referring to the waste generated at physiotherapy workplaces. One participant suggested that, for example, “each clinic could ensure it doesn't use unnecessary amounts of paper, polluting cleaning products, sorts waste, etc.”. Finally, education was also frequently mentioned, mainly on dietary choices and eco-friendly lifestyles but also without further specification.

In addition, several participants took the time to respond even if they did not provide concrete examples. Their responses commonly reflected a belief in the profession's ability and responsibility to make a positive impact, as two following quotes describe:

“Everyone need to make choices, and physiotherapists are often in key positions to influence their clients' choices and their own.”

“If there is something we can do in our work or around our work that positively impacts climate change … and everyone or most people participate, physiotherapists can certainly make an impact.”

### 3.3 Environmental behavior

Just over a quarter reported that there was no environmental policy at their workplace, and nearly a quarter were unaware of whether an environmental policy existed at their workplace or not (Table 3). A total of 74.8% of participants (*n* = 88) reported that they strive to limit environmental impacts in their professional work while 95.4% (*n* = 103) stated that they do the same as their personal choice (*p* < 0.001).

**Table 3:** Statements on environmental policy and behavior.

**Statement**		**Number (%)**
My workplace has an environmental policy	Yes	55 (50.9%)
	No	29 (26.9%)
	I don't know	24 (22.2%)
I strive to limit my environmental impacts in my work	Yes	77 (74.8%)
	No	26 (25.2%)
I personally strive to limit my environmental impacts	Yes	103 (95.4%)
	No	5 (4.6%)

**Figure 3:**
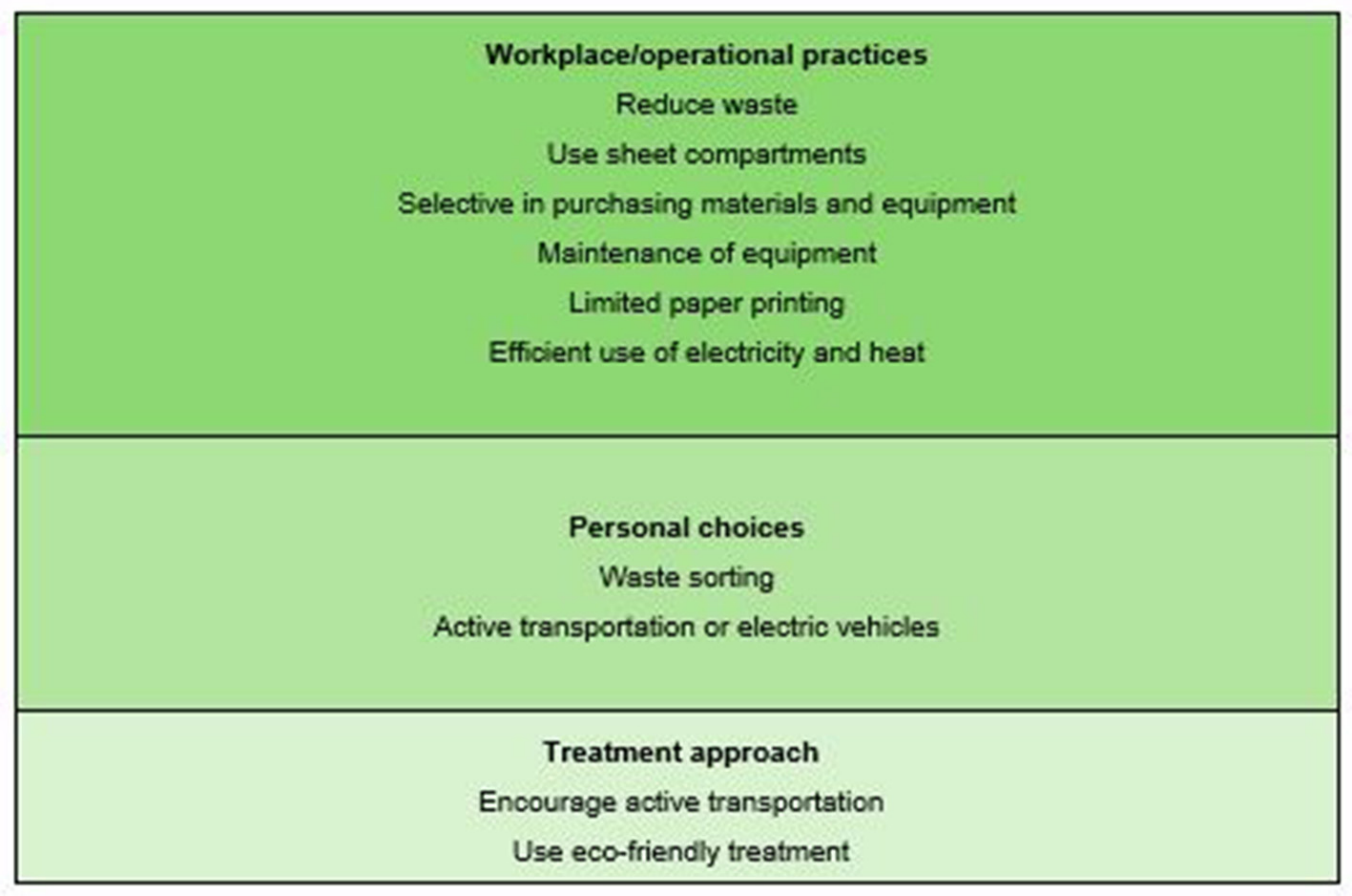
Ways to limit environmental impacts of physiotherapy.

Those who answered that they strive to limit environmental impact were given the opportunity to provide examples, and 63 participants took advantage of this. Three themes emerged from their responses (Figure 3).

Workplace/Operational practice was the most frequently mentioned theme and included various efforts to reduce waste. Participants described limiting the use of disposable items and packaging (such as paper towels, disposable sheets, plastic, and single-use packaging), reusing materials, conserving printing paper, and using energy, heating, and water efficiently. Many participants mentioned using sheet compartments to reduce laundry needs. Additionally, choices around purchasing, equipment use, and material selection were highlighted. For example, one participant stated:

“I use materials and equipment sparingly, encourage clients who need frequent or regular appointments to reuse the same sheet without washing it after each session, and maintain equipment so it lasts longer.”

Personal choice: This theme included two sub-themes. The first was waste sorting, mentioned in over half of all responses, and the second was transportation where participants reported using active transportation or electric vehicles themselves. One participant shared:

“I take the bus to and from work instead of using a private car, avoid plastic or reuse plastic bags, use stairs instead of elevators, and make full use of items (don't discard anything unnecessarily).”

Treatment approach was the least mentioned theme but advising clients on a healthy lifestyle and encouraging active transportation was mentioned. Two participants highlighted their choice of treatment methods, favoring exercise and education as primary treatment options over the use of electric devices or disposable equipment such as PEP devices.

### 3.4 Barriers and motivators for implementing eco-friendly practices

When participants were asked what they believed was potentially hindering them from adopting more eco-friendly practices in their work, the most selected response was lack of knowledge, followed closely by lack of material resources (Table 4). Guidelines was the most frequently selected factor when participants were asked about what could make it easier for them to implement eco-friendly practices in their work. Education and a formal environmental policy from their workplace or professional association followed closely.

**Table 4:** Barriers and motivators for implementing eco-friendly physiotherapy workplaces.

**Barriers**	***N* (%)**	**Motivators**	***N* (%)**
Lack of knowledge	57 (50%)	Guidelines	69 (60.5%)
Shortage of eco-friendly material/equipment	35 (30.7%)	Education on the relation between climate change, health and physiotherapy	53 (46.5%)
Lack of financial resources	22 (19.3%)	Formal environmental policy in workplace	53 (46.5%)
No environmental policy	19 (16.7%)	Formal environmental policy from IPA	52 (45.6%)
Lack of support from supervisors	13 (11.4%)	Support and encouragement from colleagues	47 (41.2%)
Too time-consuming	10 (8.8%)	Support and encouragement from supervisors	47 (41.2%)
Too much work	8 (7%)	Education resources for clients	30 (26.3%)
Lack of interest	3 (2.6%)	Evidence-based scientific articles	27 (23.7%)

IPA, Icelandic Physiotherapy Association.

## 4 Discussion

The aim of this study was to explore whether practicing physiotherapists in Iceland have knowledge and interest in climate issues and their connections to physiotherapy and health, as well as to assess their attitudes, interests, and practices in this context. The results showed that although most participants rated their knowledge as intermediate, the majority agreed that climate change affects people's health, both within and outside of Iceland. Significantly more participants stated that they try to limit their environmental impact as their personal choice, than in their professional work and lack of knowledge and resources seem to be the most hindering factors for eco-friendly practices.

### 4.1 Knowledge of climate change and relation to health

Studies among healthcare professionals have shown a reasonable general knowledge of climate change, its consequences, and its effects on human health (10–14). The findings of our study appear to align with these results. Generally, the physiotherapists seemed aware of climate change and its impact on health, and basic understanding of the issue was evident in responses to open-ended questions. Icelanders perceive their own society to be at lower risk from the effects of climate change than other societies globally (17). This perspective is also reflected in our results, where more participants believe that climate change affects health outside Iceland than within the country.

Air pollution already has significant health impacts in Europe (18) and our participants seemed aware of this as many of them mentioned respiratory illnesses when asked about the health impacts of climate change. True to the physiotherapy profession, outdoor exercise was also top of mind for many, which is a less common theme in studies among healthcare professionals. This indicates the importance of exploring the unique perspective of physiotherapists and their role in reducing the climate impact of healthcare.

Interestingly, some participants listed diverse consequences of climate change, such as weather changes, pollution, and changes in biodiversity, without explaining how these factors impacted health. A few participants also mentioned changes in the spread and frequency of specific diseases without detailing how they related to climate change. These findings suggest that while participants recognize the health risks associated with climate change and can identify potential health impacts, their understanding of the underlying mechanisms may be limited. This highlights the need to enhance the education of future physiotherapists on the relationship between climate change and health outcomes.

### 4.2 Attitudes toward climate change and role of physiotherapists

In a study by Sambath et al. (12) among healthcare professionals in India, 43.9% of respondents expressed interest in learning more about the role of healthcare professionals in the context of climate change while 87% of participants in our study were interested in learning how they could influence climate change through their work. In a recent survey on the Icelandic public (17), 64% of participants expressed concern about climate change and environmental issues but in our study, over 80% of participants reporting concerns about climate change. One possible reason for the high level of concern in our study, is that women in Iceland seem to be more concerned about these issues (17) and most of our participants were women.

Most participants agreed that it was necessary to reduce the environmental impact of physiotherapy services and healthcare in general and nearly 80% indicated that physiotherapists could have an impact on climate change within their field. Stanhope et al. (6) pointed out that educating patients to take advantage of green spaces and natural environments can enhance treatment outcomes by reducing pain perception and medication use. They also suggest that physiotherapists can play an active role in public health initiatives.

The Environmental Physiotherapy Association (19) has given some recommendations on how to make workplaces more environmentally sustainable, which include reducing the workplace's carbon footprint, such as through waste sorting and selective use of materials and resources. These aspects were the most mentioned by participants in our study. Toner et al. (9) suggested that physiotherapists encourage patients to adopt active transportation as part of their treatment to enhance physical activity. Our participants also identified this opportunity, with many mentioning encouraging clients to use active transportation and incorporating physical activity as a therapeutic tool. Education was also frequently mentioned; however, as previously noted, the content of this education was often not specified.

Physiotherapy is a noninvasive discipline, and it has been stated that physiotherapy is inherently eco-friendly, due to the emphasis on physical activity, touch and communication (7). Therefore, it has the potential to serve as an alternative to more resource-intensive interventions, such as medications and surgeries. Our participants identified physical activity and education as eco-friendly treatment options, and although not explicitly comparing physiotherapy to other treatments, nearly 70% agreed that physiotherapy is inherently eco-friendly. This perspective underscores the potential role of physiotherapists in reducing the environmental impact of the global healthcare system. If physiotherapy offers a more sustainable option compared to other treatments, its most significant contribution to minimizing healthcare's environmental footprint may lie in the effective practice of the profession. By promoting health, reducing the need for surgeries or medications, and fostering sustainable behaviors among clients, physiotherapists could play a pivotal role in advancing eco-friendly healthcare.

### 4.3 Environmental behavior

In a study by Kircher et al. (10) conducted among healthcare professionals in Minnesota, over a third of participants reported making no effort to reduce environmental impacts in their work, while in Lister et al.'s study (14) among healthcare workers in South Africa, 44% reported practicing sustainability in their work. Our study findings, however, show that most participants are already mindful of their environmental impact at work. Apart from some general actions, our participants mentioned using sheet compartments to save on laundry, and treatment-related recommendations and education on a healthy lifestyle, promoting active transportation, and choosing more eco-friendly treatment options.

It was interesting to observe that nearly all participants reported actively seeking to limit their environmental impact as their personal choice, a significantly higher proportion than those who said they did so at work. Moreover, nearly a quarter of respondents were unsure whether an environmental policy was in place at their workplace. This raises the question of why participants are less likely to demonstrate environmental responsibility at work compared to their personal choices. Prior studies among healthcare professionals show that a lack of knowledge about climate issues is often cited as a barrier (10, 11, 14). Therefore, it was not surprising that when asked about barriers in this study, lack of knowledge was the most frequently chosen response. Similarly, education and guidance on climate change in relation to physiotherapy and health were the most selected options when asked about potential motivators. These responses suggest that participants may want to improve their environmental efforts but are unsure how best to proceed. A lack of material resources was also frequently cited as a barrier, along with a lack of funding. This could indicate that in some cases, participants have ideas for solutions but lack the means or resources to implement them.

### 4.4 Strengths and limitations

This study is the first study to be conducted in Iceland among healthcare professionals, including physiotherapists, and their perspectives on climate changes and relation to health. Therefore, it is a foundational exploration of an unexamined topic in Iceland and a contribution to increasing awareness in research among physiotherapists elsewhere. True to the nature of foundational research, it raises more questions than it answers, highlighting opportunities for further research in this area. The main limitation of the study was the small size of the sample. Active members of the Icelandic Physiotherapy Association were 666 at the time of the survey, but only 114 responded, resulting in a response rate of 17.1%. It can be that physiotherapists who already had an interest in the topic were more likely to respond to the survey, despite efforts to appeal to all members during the study's promotion. Considering these factors, caution should be taken when generalizing the findings to all physiotherapists in Iceland. Nevertheless, the qualitative data from the open-ended questions and other results, can be useful in developing further research to gain deeper insights, capturing the unique perspective of physiotherapists in Iceland. This study is also a valuable input into global research in environmental physiotherapy.

## 5 Conclusions

This study is the first of its kind in Iceland to specifically highlight the perspective of physiotherapists, laying the foundation for ongoing research on climate change in relation to physiotherapy and health. The unique perspective of physiotherapists came through in the responses, revealing various opportunities connected to physical activity and outdoor engagement that have not been mentioned in other studies involving healthcare professionals. Call for guidelines on environmental physiotherapy and support for eco-friendly practice is evident. Further research should continue to explore this viewpoint, examining what role physiotherapists could play in public education and policy, what resources they feel are needed, and how they would prefer to increase their knowledge in this area.

## Data Availability

The raw data supporting the conclusions of this article will be made available by the authors, without undue reservation.
