# Supplementary material for: Environmental physiotherapy: knowledge, attitudes, and practices among physiotherapists in Iceland
Source: Front Public Health. 2025 Feb 13;13:1528217. doi: 10.3389/fpubh.2025.1528217 (PMC11865207; doi:10.3389/fpubh.2025.1528217)
Supplement: Supplementary file 1 [file Table_1.docx]

Supplementary Material

# Survey Questions

1. Do you work as a physiotherapist in Iceland?
   1. Yes (this also refers to those on temporary leave from work, such as due to maternity leave or illness)
   2. No
2. What is your gender?
   1. Female
   2. Male
   3. Non-binary
3. What is your age?
   1. Younger than 25 years old
   2. 25-35 years
   3. 36-45 years
   4. 46-55 years
   5. 56-65 years
   6. 66-75 years
   7. Older 75 years
4. What is your main workplace setting?
   1. Physiotherapist in a private clinic
   2. Physiotherapist in an institution
   3. Teaching/research
   4. Other
5. Where is your workplace located?
   1. In the capital area
   2. Outside the capital area
6. What is your knowledge of climate issues??
   1. Advanced
   2. Rather advanced
   3. Intermediate
   4. Rather little
   5. Very little
7. Do you believe that climate change and its consequences have, either directly or indirectly, an impact on people's health in Iceland?
   1. No
   2. Yes
8. Do you believe that climate change and its consequences have, either directly or indirectly, an impact on people's health outside of Iceland?
   1. No
   2. Yes
9. Can you provide an example of how you believe climate change affects people's health?
10. Please mark the option that best describes your attitude

|  | Strongly agree | Agree | Neither agree nor disagree | Disagree | Strongly disagree |
| --- | --- | --- | --- | --- | --- |
| It is necessary to limit the negative impact of healthcare services on the environment | ❏ | ❏ | ❏ | ❏ | ❏ |
| The negative impact of physiotherapy services on the environment must be limited | ❏ | ❏ | ❏ | ❏ | ❏ |
| I am concerned about climate change | ❏ | ❏ | ❏ | ❏ | ❏ |
| I am interested in learning more how I can reduce environmental impact in my work | ❏ | ❏ | ❏ | ❏ | ❏ |
| Physiotherapists should raise public awareness of the health impacts of climate change | ❏ | ❏ | ❏ | ❏ | ❏ |
| Physiotherapists should raise their clients' awareness of the health impacts of climate change | ❏ | ❏ | ❏ | ❏ | ❏ |
| Physiotherapy is inherently eco-friendly | ❏ | ❏ | ❏ | ❏ | ❏ |

1. Do you believe that physiotherapists can have an impact on climate change (negative or positive) within their area of work
   1. No
   2. Yes

- If yes, can you provide example(s)?: _______________________

1. There is an environmental policy at my workplace
   1. Yes
   2. No
   3. I don´t know
2. Do you strive to limit environmental impact in your work?
   1. No
   2. Yes

- If yes, can you provide example(s)?: _______________________

1. Do you personally strive to limit environmental impact?
   1. No
   2. Yes
2. Please select the factors (one or more) that you believe hinder you from implementing more environmentally friendly practices in your work.
   1. Lack of financial resources
   2. Lack of support from supervisors
   3. Shortage of eco-friendly material/equipment (e.g. types of environmentally friendly equipment, tools, facilities, clothing, materials, or other goods)
   4. Lack of knowledge
   5. There is no environmental policy at my workplace
   6. It is too time-consuming
   7. It is too much work
   8. I am not interested
   9. Other, what? _________________________
3. Please select the factors (one or more) that make it easier for you, or could make it easier for you, to implement more environmentally friendly practices in your work.
   1. Formal environmental policy in workplace
   2. Formal environmental policy from IPA
   3. Support and encouragement from supervisors
   4. Support and encouragement from colleagues
   5. Guidelines on more eco-friendly practice in physiotherapy
   6. Education (such as lectures, written educational materials, courses, or instructional videos) regarding the connections between climate change, physiotherapy, and health
   7. Education resources for clients
   8. Access to evidence-based scientific articles
   9. I am not interested in implementing more environmentally friendly practices in my work.
   10. Other, what? _________________________
